# Neurophysiological signals as predictive translational biomarkers for Alzheimer’s disease treatment: effects of donepezil on neuronal network oscillations in TgF344-AD rats

**DOI:** 10.1186/s13195-018-0433-4

**Published:** 2018-10-10

**Authors:** Milan Stoiljkovic, Craig Kelley, Tamas L. Horvath, Mihály Hajós

**Affiliations:** Translational Neuropharmacology, Department of Comparative Medicine, Yale University School of Medicine, 310 Cedar St, New Haven, CT 06520 USA

**Keywords:** Alzheimer’s disease, TgF344-AD rats, Hippocampus, Theta, Phase-amplitude coupling, High-voltage spindles, Donepezil

## Abstract

**Background:**

Translational research in Alzheimer’s disease (AD) pathology provides evidence that accumulation of amyloid-β and hyperphosphorylated tau, neuropathological hallmarks of AD, is associated with complex disturbances in synaptic and neuronal function leading to oscillatory abnormalities in the neuronal networks that support memory and cognition. Accordingly, our recent study on transgenic TgF344-AD rats modeling AD showed an age-dependent reduction of stimulation-induced oscillations in the hippocampus, and disrupted long-range connectivity together with enhanced neuronal excitability in the cortex, reflected in greatly increased expression of high-voltage spindles, an epileptic absence seizure-like activity. To better understand the translational value of observed oscillatory abnormalities in these rats, we examine here the effects of donepezil, an acetylcholine esterase inhibitor clinically approved for AD treatment.

**Methods:**

Brainstem nucleus pontis oralis stimulation-induced hippocampal oscillations were recorded under urethane anesthesia in adult (6-month-old) and aged (12-month-old) TgF344-AD and wild-type rats. Spontaneous cortical activity was monitored in a cohort of freely behaving aged rats implanted with frontal and occipital cortical electroencephalography (EEG) electrodes.

**Results:**

Subcutaneous administration of donepezil significantly augmented stimulation-induced hippocampal theta oscillation in aged wild-type rats and both adult and aged TgF344-AD rats, which have been previously shown to have diminished response to nucleus pontis oralis stimulation. Moreover, in adult TgF344-AD rats, donepezil also significantly increased theta phase-gamma amplitude coupling in the hippocampus during stimulation. However, neither of these effects were significantly changed in adult wild-type rats. Under freely behaving conditions, donepezil treatment had the opposite effect on cortical oscillatory connectivity in TgF344-AD and wild-type rats, and it reduced the occurrence of high-voltage spindle activity in TgF344-AD rats.

**Conclusions:**

Together, these results imply that pharmacologically enhancing cholinergic tone with donepezil could partially reverse oscillatory abnormalities in TgF344-AD rats, which is in line with its clinical effectiveness in AD patients. Therefore, our study suggests good translational opportunities for these neurophysiological signals recorded in TgF344-AD rats, and their application could be considered in drug discovery efforts for developing therapies with disease-modifying potential.

## Background

Progress made in the development of new and more effective treatments for Alzheimer’s disease (AD), a devastating neurodegenerative disorder, is disappointing since many new compounds, despite initial promise at the preclinical level, fail in clinical trials [1]. A significant setback partly lies in our still insufficient understanding of the complex pathophysiology of AD, ultimately causing lack of tractable biomarkers that would reliably measure disease progression and response to therapy. Another factor hampering successful translation from bench to bedside is that the transgenic animal models of AD used in the early phases of drug discovery, though capturing some pathological aspects of disease (e.g., amyloid plaques and neurofibrillary tangles), often do not faithfully simulate critical interactions between processes underlying aging and the development of AD traits observed in humans [2]. Given this disconnect between preclinical and clinical data, back-translational studies using clinically relevant measures and currently available therapies are urgently needed for evaluating the predictive validity of animal AD models to ensure their further use in the drug discovery process [3].

In recent years, electroencephalography (EEG) methodologies have emerged as useful tools for screening novel therapeutics. With its applicability in different species, high temporal resolution, and similarities in read-outs from animals and humans, EEG offers the promise to provide surrogate measures of drug efficacy and also to predict the impact of developmental compounds on endophenotypes associated with the disease in both clinical and preclinical settings [4, 5]. Moreover, the relation of EEG measures in particular brain regions to certain functions, such as cognition, is suggested to be consistent between animals and humans [6–9]. In accordance, abnormality of hippocampal and cortical EEG rhythms in AD and related dementias has been linked to altered cognitive functions in both patients and transgenic rodents [10–12], indicating the potential of EEG for use as a putative translatable biomarker for diseases with impaired cognition. In our recent study on transgenic TgF344-AD rats modeling AD [13], we reported a number of EEG abnormalities in the hippocampal and cortical oscillatory networks, with some preceding and others paralleling impairment in their cognitive functions [14]. Specifically, we identified an age-dependent reduction of stimulation-induced hippocampal theta oscillation and decreased theta phase-gamma amplitude coupling in TgF344-AD rats when compared with their wild-type (WT) counterparts. We also found disrupted intercortical and hippocampal-cortical long-range oscillatory connectivity in TgF344-AD rats, which align with findings from a concurrent study where magnetic resonance imaging-based connectomics analysis showed correlation between cognitive impairments and disruption in connectivity of structural and functional networks in memory-related regions in these rats at the early stage of AD-related pathology [15]. Furthermore, in TgF344-AD rats, cortical EEG revealed enhanced neuronal excitability with significantly increased expression of high-voltage spindles (HVSs), resembling epileptic absence seizure-like activity. Along with these changes, TgF344-AD rats exhibited impairments in sensory information processing as revealed by tests which are also used in clinical practice [13]. Importantly, many of these functional abnormalities observed in TgF344-AD rats mirror alterations observed in AD patients [10, 16, 17].

Based on these neurophysiological observations, and the presence of several AD-related pathologies in TgF344-AD rats including age-dependent amyloidosis, tauopathy, apoptotic loss of neurons, as well as cognitive disturbance [14], this transgenic rodent model seems to be suitable for translational research. Therefore, in this study we examined the effects of donepezil, an acetylcholine esterase inhibitor clinically approved for treatment of AD, on neuronal network oscillations in TgF344-AD rats to evaluate the potential of neurophysiological signals as predictive translational biomarkers for AD treatment.

## Methods

### Animals

Heterozygous TgF344-AD rats (Rat Resource and Research Center, Columbia, MO) and their age-matched wild-type (WT) Fischer344 counterparts (Envigo, Indianapolis, IN) were used in this study. TgF344-AD rats were generated onto Fisher344 strain by co-injection of two transgenes both driven by mouse prion promoter elements: one contains the human Aβ precursor protein with the Swedish mutation (APPswe), and the other contains the human presenilin 1 with a deletion of exon 9 (PS1ΔE9). A total of 32 male TgF344-AD and WT rats were analyzed at two age points: adult (6 months old; *n* = 6 for each genotype) and aged (12 months old, *n* = 10 for each genotype) rats. Before being used in experiments, animals were housed in a temperature and humidity-controlled room with a 12:12-h light-dark cycle and with free access to food and water at all times. During the study, all measures were taken to minimize pain or discomfort of the rats. All procedures were performed according to the protocol reviewed and approved by the Yale University Institutional Animal Care and Use Committee and in compliance with the NIH Guide for the Care and Use of Laboratory Animals (NIH Publications No. 80–23, revised 1996).

### Electrophysiology

Recordings were carried out in anesthetized and in freely behaving rats. For experiments under anesthesia, adult (TgF344-AD = 6; WT = 6) and aged (TgF344-AD = 5; WT = 5) rats were injected with urethane (1.5 g/kg) intraperitoneally and placed in a Kopf stereotaxic frame (Tujunga, CA) on a temperature-regulated heating pad (Physitemp Instruments Inc., Clifton, NJ) set to maintain body temperature at 37–38 °C. Following unilateral craniotomies above the hippocampus and rostral pons, acute electrodes were inserted for recording of local field potentials (LFPs) from hippocampal CA1 region and for stimulation of nucleus pontis oralis (nPO). LFPs were recorded using a 16-site silicon recording electrode (A1 × 16–10 mm-100–177-T15; NeuroNexus Technologies, Inc., Ann Arbor, MI) positioned 3.0 mm posterior and 2.0 mm lateral to bregma, and slowly lowered 2.5–3.0 mm from the cortical surface to span the dorsal hippocampus. For electrical stimulation of the nPO, a bipolar concentric stainless steel electrode (NE-100X, Rhodes Medical Instruments, Woodland Hills, CA) was placed 7.8 mm posterior and 1.8 mm lateral to bregma, and 6.0 mm ventral from the cortical surface. All stereotaxic coordinates were taken from the atlas of Paxinos and Watson [18]. An ear bar of the stereotaxic frame served as the ground. Prior to recordings, each rat was allowed to stabilize for at least 30 min. The nPO stimulation, consisting of a train of 0.3-ms square pulses at 250 Hz over 6 s, was delivered every 100 s by an Isoflex stimulus-isolator (A.M.P.I. Instruments, Jerusalem, Israel). The stimulating current was increased stepwise from 0.0 to 0.2 mA in 0.02-mA increments and repeated in two cycles to establish a stimulus-response relationship for both total power and peak frequency in the theta band. Throughout the recordings, rats were kept in the stereotaxic frame, spontaneous and stimulation-induced hippocampal LFPs were continuously monitored, and the level of anesthesia regularly checked.

Experiments under freely behaving conditions were performed in another cohort of aged rats (TgF344-AD = 5; WT = 5) implanted with chronically indwelling electrodes. After achieving a surgical plane of anesthesia (mixture of ketamine 60 mg/kg and xylazine 10 mg/kg, given intraperitoneally), rats were placed in a stereotaxic frame, their skin shaved and cleaned, and the skull exposed. Stainless steel monopolar screw electrodes were inserted under aseptic conditions over the frontal (1.0 mm anterior and 2.0 mm lateral to bregma), and occipital (6.5 mm posterior and 3 mm lateral to bregma) cortices. To serve as a ground and reference, an additional two screws were placed over the cerebellum. All electrodes were joined to a miniature connector which was fixed to the skull using dental acrylic, and then the skin incision was sutured. After surgery, each rat was put in a clean cage and all necessary postoperative care was taken. Following a 10-day recovery period, recordings were carried out in groups of two rats at a time (one TgF344-AD and one WT) in their own home cages and repeated weekly during the light phase (between 10:00 and 14:00). For every recording session, rats were connected using stainless steel spring protected cables to slip-ring commutators, with their cages placed in individual recording boxes and left to accommodate for at least 30 min prior to recordings.

For data acquisition, LFPs were amplified and filtered between 1 and 300 Hz using A-M System (Carlsborg, WA, USA) with an additional notch filter at 60 Hz. The signal was simultaneously sampled at 2 kHz and stored on a computer via a CED Micro1401–3 interface and Spike2 software (Cambridge Electronic Design, Cambridge, UK). All offline filtering was performed using the EEGLAB toolbox (Delorme and Makeig 2004), and data were analyzed using custom-written Matlab (Mathworks, Natick, MA) programs. For quantitative analysis of nPO stimulation-induced hippocampal oscillations, the first second of each 6-s long stimulation period was omitted to avoid possible stimulation artifacts. Absolute theta power (3–9 Hz) and peak theta frequency were determined at each stimulation intensity and averaged over the three adjacent channels with the highest LFP amplitude for each animal. Once the stimulus intensity required to elicit theta oscillation between 60% and 80% of the maximal amplitude was determined for each rat, it was held constant for the time course of the experiment. After establishing a stable baseline for 15 min, donepezil was injected, and recording continued for another 60 min. For experiments under freely behaving conditions, 30 min of undisturbed cortical recordings were used for baseline, then donepezil was injected and effects followed during an additional 60 min of postinjection recordings.

Donepezil (donepezil hydrochloride; TCI, Tokyo, Japan) was dissolved in 0.9% sterile saline as free base equivalent and administered subcutaneously in a dose of 1 mg/kg in all experiments. The dose corresponds to the clinical dose of donepezil based on previous comparison of the exposure response relationship in rats and clinical efficacious exposure of donepezil in humans [19]. Diazepam (Sigma, St. Louis, MO) was dissolved in a 10% (2-hydroxypropyl)-β-cyclodextrin aqueous solution, and given subcutaneously in a dose of 1 mg/kg in a set of experiments where pharmacological effects on HVSs were analyzed.

### Data analysis

For analysis of elicited hippocampal theta power under anesthesia, total theta power in the 3–9 Hz frequency band during nPO stimulations was normalized for each animal to the mean power of the baseline responses measured before donepezil administration. Normalized time-course power values were then grouped according to genotype and age. Theta phase-gamma amplitude coupling during hippocampal stimulation was assessed using modulation index (MI), as described previously [20, 21]. Briefly, the phase containing signal from 3 to 12 Hz, and the amplitude containing signal from 20 to 100 Hz were used to calculate the MI value at each frequency pair for the LFPs recorded during each stimulation. To generate the heatmaps, the MI values at each frequency pair were averaged from each animal in the TgF344-AD or WT group for signal duration over nine stimulations during baseline and after donepezil, beginning 30 min following injection. Statistical comparisons of the average MI were performed for either low gamma (30–55 Hz) or high gamma (65–90 Hz) and between 4 Hz and 6 Hz in phase frequency (corresponding to the peak frequency of elicited theta oscillation) calculated for each animal at baseline as well as after donepezil injection.

Cortical spectral power density was calculated from 5 min of spontaneous EEG from each animal. Spectral density was smoothed with a moving average filter with a span of 0.18 Hz. Automatic detection of HVSs was performed using the signal from the frontal cortex when the rhythmic negative deflections lower than −0.3 mV occurred while instantaneous power between 6 and 12 Hz was more than double instantaneous delta power for 2 s or longer. The probability of occurrence of spontaneous HVSs was calculated over a 30-min period before and 30 min after treatment for each TgF344-AD and WT rat, and then compared between groups. Long-range oscillatory connectivity between frontal and occipital cortices in freely behaving rats was assessed before (baseline) and after donepezil treatment by averaging phase-locking values (PLV) [22, 23] in 20 contiguous 10-s long epochs in each animal.

All data were initially determined to be suitable for parametric analysis according to normality and homoscedasticity and grouped based on genotype and age. The effect of donepezil on stimulation-induced hippocampal theta power change was then tested using two-way mixed design analysis of variance (ANOVA) with a genotype as between-subject factor and time as within-subject factor. Subsequently, a two-tailed *t* test was performed on individual groups to examine the treatment effect related to baseline. Cortical power spectra changes were compared using two-way ANOVA. Changes in all other metrics tested were assessed with two-tailed *t* tests. Data are expressed as the mean ± standard error of the mean, and differences were considered significant when *p* < 0.05.

## Results

**Fig. 1:**
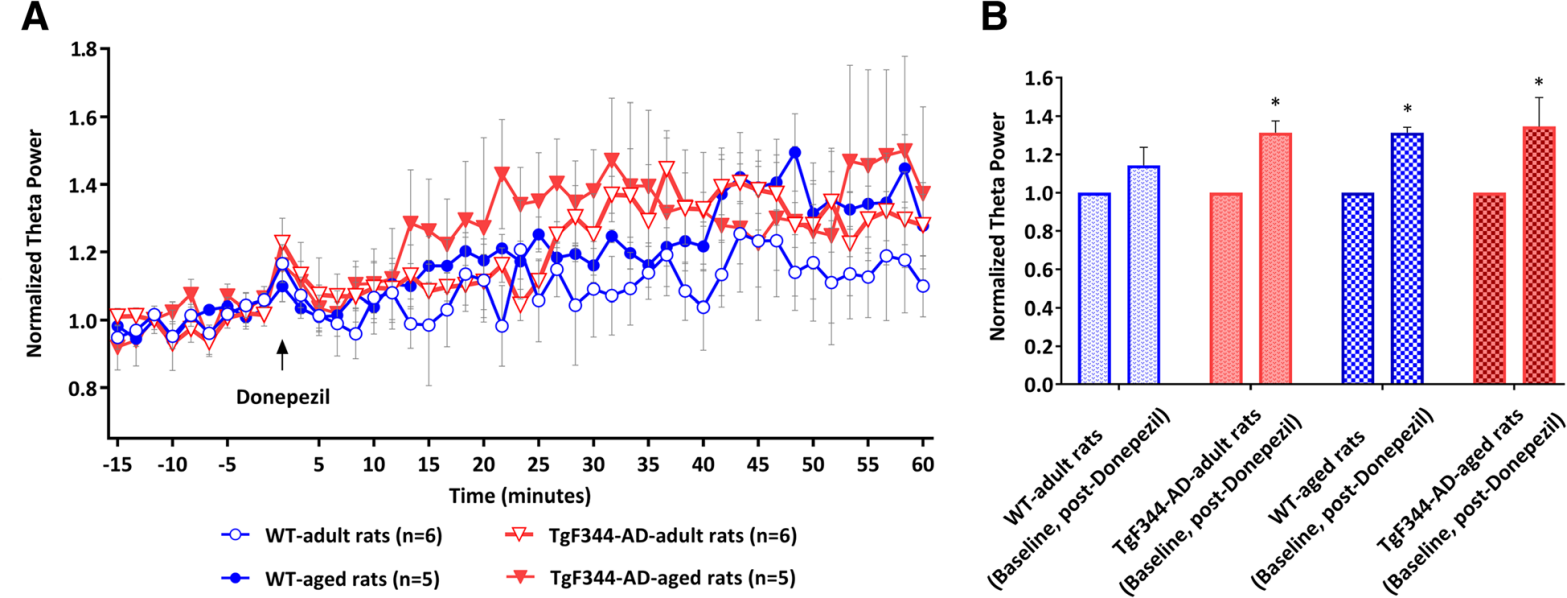
Effect of donepezil on nPO stimulation-induced theta oscillation in the hippocampus of urethane anesthetized adult and aged TgF344-AD rats and their wild-type (WT) counterparts. **a** Total theta power in the 3–9 Hz frequency band was normalized for each animal to the average power prior to injection (indicated by the arrow) and values grouped according to genotype and age. **b** Comparison of grouped theta power values obtained during baseline and 30–60 min after donepezil treatment showed a significant increase in theta power in both adult and aged TgF344-AD rats, as well as in aged WT rats (*p < 0.05, two-tailed *t* test)

High-frequency electrical stimulation of the nPO elicited hippocampal network oscillations in the theta range, with a current-dependent increase of peak frequency in both WT and TgF344-AD rats under urethane anesthesia, in line with previous findings in rats and mice [20, 24–26]. Consistent with our recent report on TgF344-AD rats [13], stimulus-response analysis showed a reduction in hippocampal oscillatory activity with a particularly diminished theta power in both adult and aged TgF344-AD rats compared with their respective WT rats. Both adult and aged WT and TgF344-AD rats similarly responded to systemic subcutaneous administration of donepezil at the dose of 1 mg/kg (F_(3,18)_ = 1.058; *p* = 0.39 two-way mixed design ANOVA), significantly increasing stimulation-induced theta power (Fig. 1a) over the course of experiment (F_(44,792)_ = 7.197; *p* = 0.0005, two-way mixed design ANOVA). Comparison of the effect of donepezil on theta power change relative to baseline revealed an increase in elicited theta in adult and aged TgF344-AD (*p* < 0.03, for each) as well as aged WT rats (*p* < 0.005), while in adult WT rats the power of theta increased, but significance was not reached (Fig. 1b). In rats where donepezil showed a significant effect, overall enhancement of theta power measured between 30 and 60 min after treatment was about 30–35% of baseline theta. Interestingly, both adult and aged TgF344-AD, as well as aged WT rats, previously showed diminished response to nPO stimulation, which implies impairment of hippocampal oscillatory function [13].

**Fig. 2:**
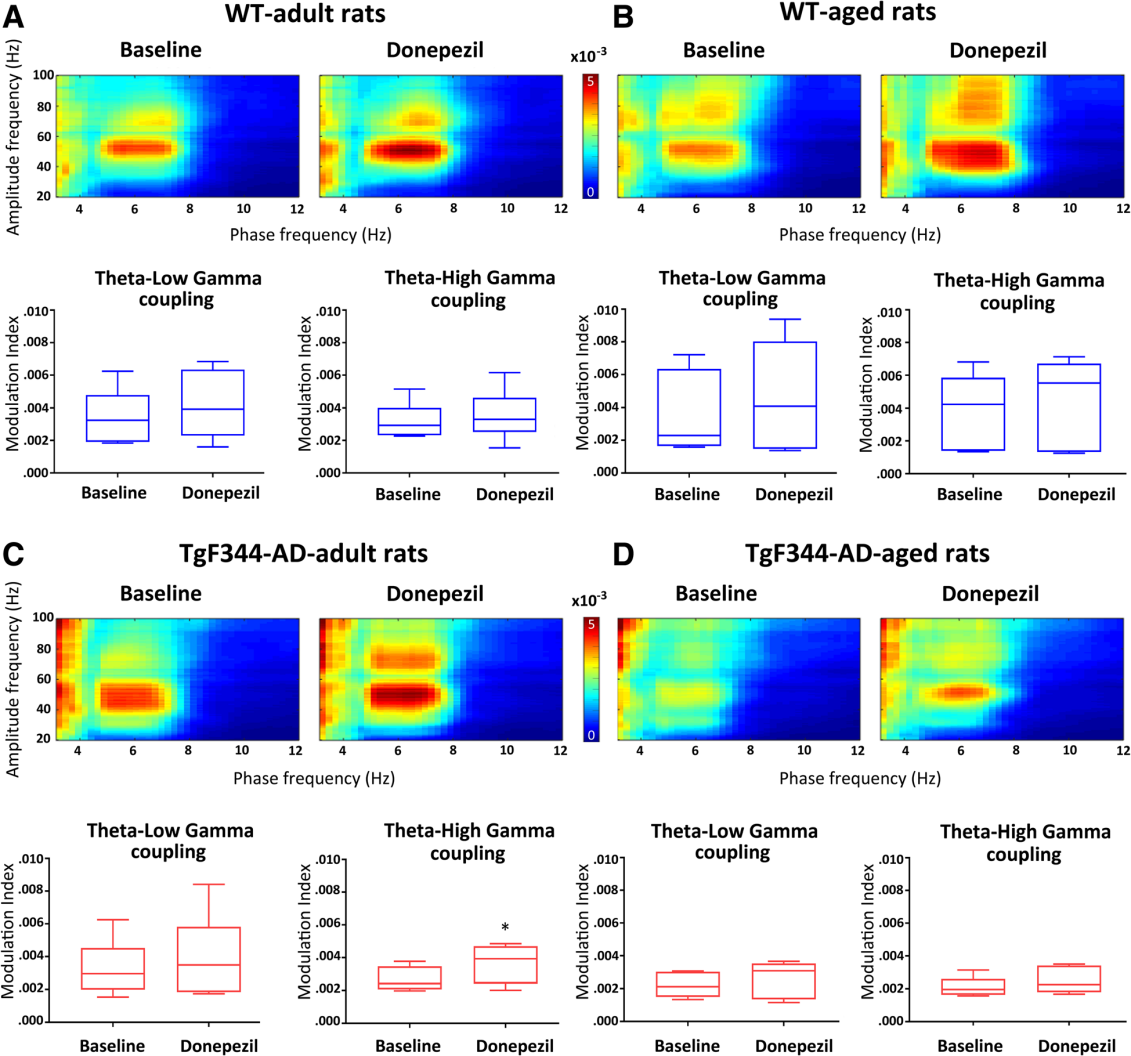
Effect of donepezil on stimulation-induced theta phase-gamma amplitude coupling in the hippocampus of urethane anesthetized wild-type (WT) and TgF344-AD rats. Theta-gamma coupling was averaged over all animals in each (**a**,**b**) WT and (**c**,**d**) TgF344-AD group at both baseline and 30 to 45 min after drug injection. The strength of theta-gamma coupling was expressed by the modulation index (MI). Although baseline theta-gamma coupling was enhanced after donepezil treatment in both WT and TgF344-AD rats at each age group, a significant increase in MI was found only in adult transgenic rats (**c**, *p < 0.05, two-tailed *t* test)

To further explore the effects of donepezil treatment on complex oscillatory dynamics in the hippocampus during stimulation, we calculated the modulation index (MI) to measure theta phase-gamma amplitude coupling in TgF344-AD and WT rats. Administration of donepezil enhanced baseline theta-gamma coupling in both TgF344-AD and WT rats at each age tested as shown in heatmaps (Fig. 2). However, an increase in strength of theta-high gamma coupling reached a significant level only in adult TgF344-AD rats (Fig. 2c, *p* < 0.05, two-tailed *t* test), in part due to the high variation within the groups.

**Fig. 3:**
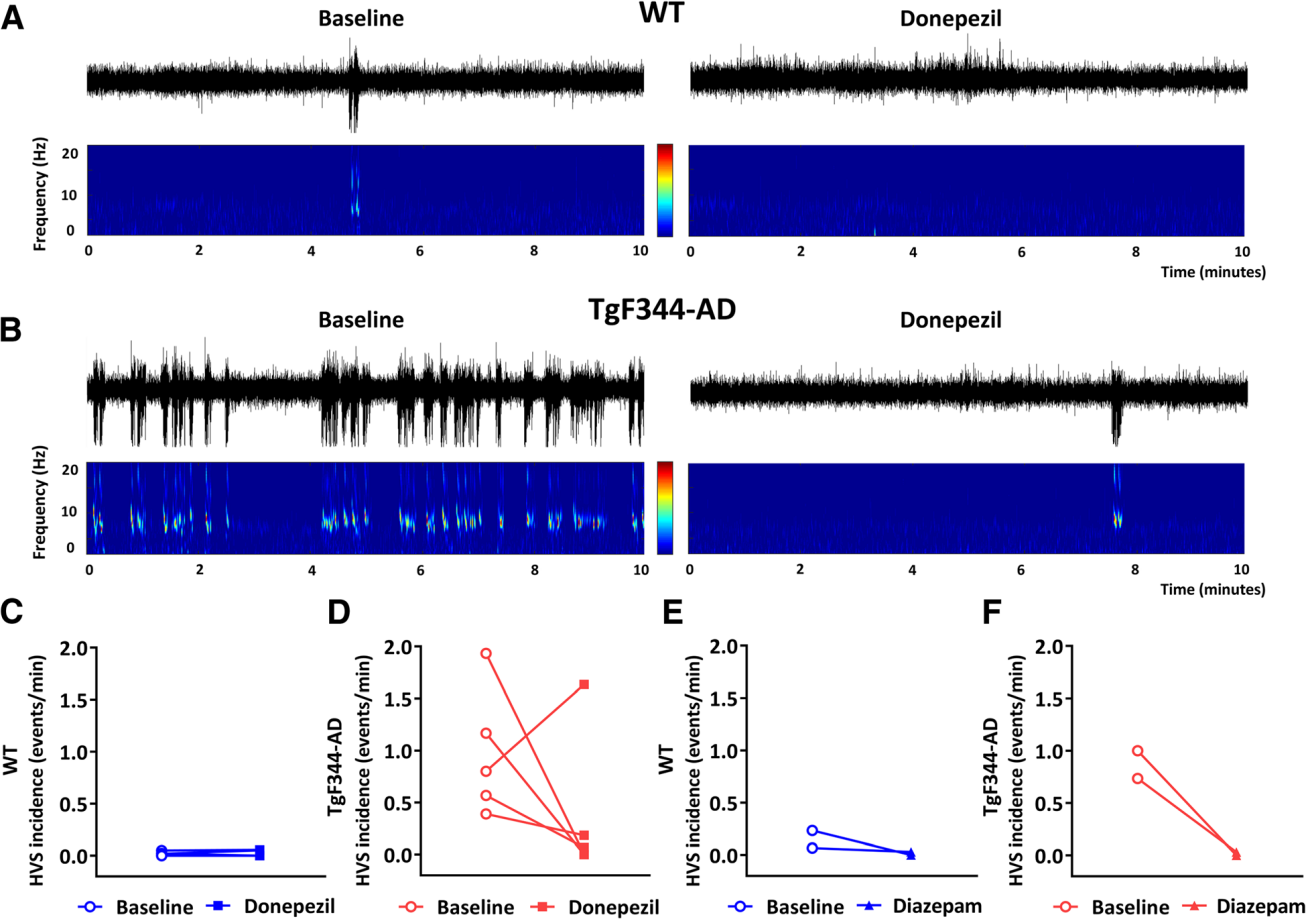
Effect of donepezil on spontaneous paroxysmal high-voltage spindles (HVSs) in the cortex of aged wild-type (WT) and TgF344-AD rats. **a**,**b** Example traces and spectrograms of 10 min of spontaneous frontal cortex EEG from WT and TgF344-AD rats at baseline and after donepezil injection. Large negative deflections in the EEG and prominent bouts of high power activity between 6 and 9 Hz in the spectrogram are HVS events. **c**,**d** Group effect of donepezil (1 mg/kg) injection on HVSs in WT and TgF344-AD rats. **e**,**f** Group effect of diazepam (1 mg/kg) injection on HVSs in WT and TgF344-AD rats

During awake but idling behavior, spontaneously occurring highly synchronous paroxysmal spike-wave discharges with 7–8 Hz peak frequency were presented in the cortical EEG of freely behaving aged TgF344-AD and WT rats, in line with our previous observation [13]. This distinct oscillatory pattern, resembling epileptic absence seizure-like activity, was defined as high-voltage spindles (HVSs) and presented with a particularly pronounced amplitude in the frontal cortex EEG where it intermittently disrupted regular cortical activity (Fig. 3a, b). In the occipital cortex EEG, HVSs were barely if at all detectable in any rat from either genotype. During baseline recordings, HVSs were identified very frequently in four TgF344-AD rats and occasionally in one TgF344-AD rat out of the recorded five rats. In contrast, only two WT rats showed HVSs and only occasionally during the course of the experiments. Systemic administration of donepezil reduced the occurrence of HVSs in four TgF344-AD rats and in one WT rat, while it did not affect HVS occurrence in one TgF344-AD and one WT rat (Fig. 3c, d). For further exploration of the property of HVSs and their ability for pharmacological modulation, we treated a subset of animals (TgF344-AD = 2; WT = 2) expressing HVSs at baseline with diazepam which is used to treat patients with epileptic seizures [27]. In these rats, diazepam completely eliminated the occurrence of HVSs (Fig. 3e, f).

**Fig. 4:**
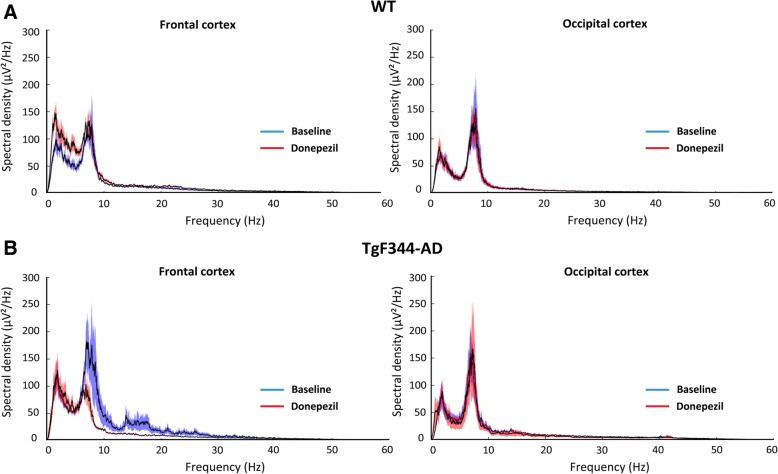
Power spectral density of spontaneous cortical EEGs in aged wild-type (WT) and TgF344-AD rats. Donepezil significantly increases spectral density (F_(3.1 × 10–5,2.5 × 10–5)_ = 1.88; *p* < 0.0001, two-way ANOVA) in the WT frontal cortex (**a**), and significantly decreases spectral density (F_(3.1 × 10–5,2.5 × 10–5)_ = 1.81; *p* < 0.0001, two-way ANOVA) in the TgF344-AD frontal cortex (**b**). Donepezil has no significant impact on spectral density in the occipital cortex for either WT or TgF344-AD

**Fig. 5:**
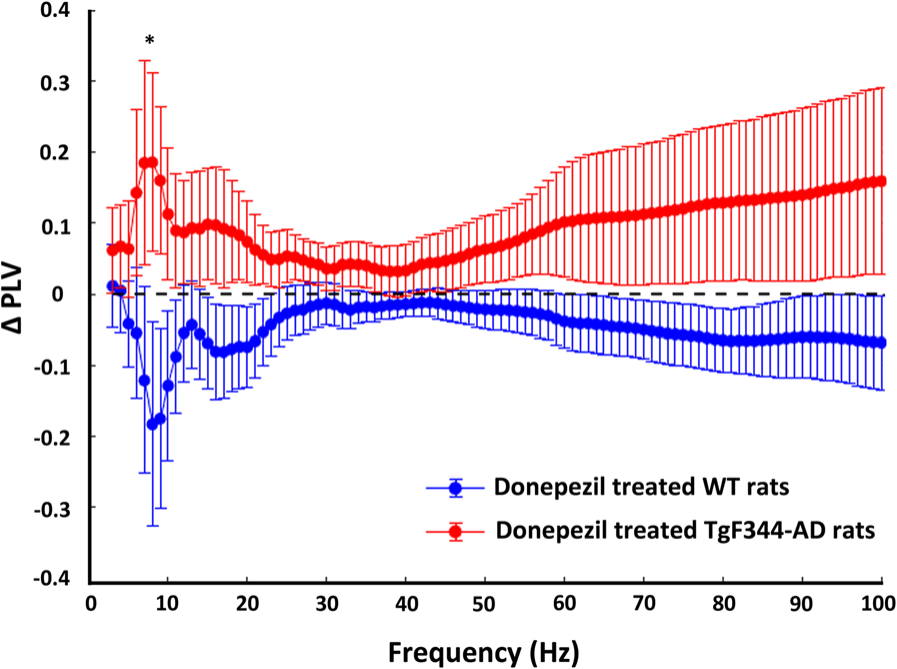
Change in occipital-frontal phase locking in response to donepezil treatment. Donepezil on average increases phase locking between occipital cortex and frontal cortex in TgF344-AD rats and decreases phase locking in wild-type (WT) rats. The magnitude of the change in phase-locking values (PLV) from baseline to post-donepezil injection is significant at theta frequencies (*p < 0.05, two-tailed *t* test)

Donepezil had differential effects on both cortical spectral density and frontal-occipital phase locking in aged rats. In TgF344-AD rats, donepezil significantly decreased spectral density in the frontal cortex (F_(3.1 × 10–5,2.5 × 10–5)_ = 1.88; *p* < 0.0001, two-way ANOVA), particularly in the 7–9 Hz and 14–18 Hz frequency ranges. Conversely, donepezil significantly increased spectral density in the frontal cortex of WT rats (F_(3.1 × 10–5,2.5 × 10–5)_ = 1.81; *p* < 0.0001, two-way ANOVA), particularly in the theta frequency range (Fig. 4). Meanwhile, donepezil increased PLV in TgF344-AD rats and decreased PLV in WT rats. The magnitude of the change in PLV was significantly different in the theta range (*p* < 0.05, two-tailed *t* test), but on average was divergent across frequency bands (Fig. 5).

## Discussion

The value of neurophysiological signals associated with AD for predicting treatment outcomes has not been fully explored and is not generally agreed upon. Here, we studied the effects of donepezil, currently a standard therapy for patients with AD, on a number of EEG abnormalities detected in TgF344-AD rats in an attempt to validate neurophysiological signals recorded in these rats for application in the development of new AD therapies. We found that acute systemic administration of donepezil in TgF344-AD rats increased stimulation-induced hippocampal theta oscillation power (both in adult and aged rats) and theta phase-gamma amplitude coupling (in adult rats) under anesthesia. Donepezil also reduced cortical hyperexcitability under freely behaving conditions, and increased frontal-occipital phase locking in TgF344-AD rats, whereas it showed the opposite effect in their WT counterparts. These results are particularly important since this is the first neurophysiological study on donepezil in a transgenic AD model that contains both amyloid plaques and hyperphosphorylated tau [14], allowing us to determine some of its effects under pathologies paralleling human AD that cannot be readily examined in patients with conventional EEG recording.

Ample evidence suggests a close association between the abnormalities in neuronal oscillations within the cortex and hippocampus and the impairment in cognitive functions presented in neurodegenerative disorders including AD (reviewed in [16, 28]). Accordingly, generalized slowing of the EEG with power spectral profile shift to the low frequencies has been linked to cognitive deterioration in AD patients [29]. At the preclinical level, multiple alterations in both cortical and hippocampal networks have been identified in several transgenic AD mouse models which demonstrate learning and memory deficiencies [30, 31]. Importantly, distinctive hippocampal oscillatory abnormalities, such as reduction in theta band power and weakening of theta-gamma coupling, have been shown to precede cognitive deficits and emergence of Aβ plaques and neurofibrillary tangles, and reflect disease progression [20, 24, 25, 32]. Theta oscillation is found to be the most prominent activity in the hippocampus during learning, memory, and exploratory behavior [6, 9], and correlation between hippocampal theta and cognitive processes has been indicated in both experimental animals [6] and humans [7, 33]. Furthermore, coupling between theta and gamma oscillations (theta phase-gamma amplitude coupling) in the hippocampus has been shown to implicate effective memory encoding and consolidation both in patients (implanted with depth electrodes) and rodents [21, 34]. Based on these findings, it has been proposed that pharmacological modulation of hippocampal theta oscillation could serve as a quantitative measure to predict the cognitive effects of drugs. Therefore, an in-vivo assay to track hippocampal theta changes independent of behavior that involves nPO stimulation-induced theta oscillation in anesthetized animals has been developed [35]. Using this assay, it was shown that drugs with procognitive potential increase hippocampal theta power, while drugs that exhibit deleterious effects on hippocampal-dependent cognitive functions, such as muscarinic and NMDA receptor antagonists, diminish theta power (reviewed in [35, 36]).

In our study, donepezil significantly increased stimulation-induced hippocampal theta power in both adult and aged TgF344-AD rats as well as aged WT rats, which were previously shown to have diminished theta power in response to nPO stimulation [13]. Furthermore, donepezil showed a trend towards increased theta phase-gamma amplitude coupling in the hippocampus during stimulation both in WT and TgF344-AD rats; however, coupling strength significantly increased only in adult TgF344-AD rats. Hippocampal theta oscillation is mainly regulated by coordinated output of the cholinergic and GABAergic neurons, located in the medial septum/diagonal band of the Broca complex (MS/DB) of the basal forebrain (BF), which provides a rhythmic drive to the hippocampal pyramidal cells for firing in theta frequency [6, 37]. Donepezil, by increasing acetylcholine levels and cholinergic signaling in the MS/DB-to-hippocampus network [38], likely amplifies synchronous firing of pyramidal cells and thus enhances hippocampal theta power and theta phase-gamma amplitude coupling as found in the current study. Interestingly, our results showed that acute donepezil administration efficiently increased stimulation-induced theta power only in transgenic rats and aged WT rats, but not in adult WT rats. This could be explained by the possible difference in baseline cholinergic function between these rats. In fact, a reduction in cholinergic neuronal functions in the BF region has been observed in AD but also over the course of aging [39, 40]. Meanwhile, it is expected that the cholinergic system in healthy adult subjects is fully functional, thereby limiting the potential for pharmacological manipulation of cholinergic neurotransmission with donepezil. In support of this premise, previous studies demonstrated that donepezil treatment has no effects on a range of cognitive functions, including attention and working memory, in adult healthy humans [41, 42].

A recent clinical study reported a high incidence of unprovoked nonconvulsive seizures in patients with AD monitored by overnight long-term video EEG [17], indicating that this absence-like epileptiform activity may be more common in AD than previously recognized. Furthermore, it has been suggested that these absence seizures, which are indicative of neuronal hyperexcitability, might contribute to accelerated cognitive decline in AD [43]. Spontaneous nonconvulsive seizures were also described in different lines of transgenic AD mice, and a correlation between these aberrant oscillations and impairments in cognitive performances was established [44, 45]. Consistent with these reports and our recent study [13] in the cortical EEG of freely-behaving aged TgF344-AD rats, we observed high expression of paroxysmal HVSs, hypersynchronous oscillatory bursts occurring during passive wakefulness. These events were also noted, although very rarely, in some age-matched WT rats, similar to previous observation of HVSs in aged rats of several strains [46, 47]. HVSs are considered to reflect epileptic absence seizures given their spike and wave pattern, unresponsiveness to mild stimuli, and their reduction by antiepileptic drugs [48]. Importantly, it has been shown that an increased occurrence of HVSs in rats coincided with their decline in spatial memory performance [47], which accordingly could explain the spatial memory deficits in TgF344-AD rats previously reported [14].

In this study we showed that treatment with donepezil had a clear tendency towards a reduction of HVSs in both TgF344-AD and WT rats. This modulatory effect of donepezil is due to an increase in cholinergic tone, since studies have suggested cholinergic depletion in the nucleus basalis (NB) of BF and thalamocortical dysfunction as the likely factors for HVS induction [46, 49]. Specifically, cholinergic projections of NB provide both activation of the neocortex [40] and inhibition of the reticular nucleus of the thalamus (RT) [46]; therefore, reduction of BF cholinergic tone may lead to impairment of cortical activation, and disinhibition of RT and thalamocortical projections, which trigger nonconvulsive HVS discharges in the neocortex. In that regard, these spontaneous absence seizure-like epileptiform discharges in the cortical EEG may serve as an indicator of declining NB cholinergic function in AD or during the aging process.

The reduction in hypersynchronous HVSs in the cortex with donepezil coincided with normalization in the power spectral profile in TgF344-AD rats to the level of the baseline WT power spectrum, which further indicates a possible improvement in cortical network functions and cognitive processing. It is believed that frequent absence seizures may heavily affect attention, learning, and the ability to process information as they cause paroxysmal disruptions of cortical network activity followed by brief lapses of consciousness reflected in inattention and vacant staring [50]. Furthermore, in AD patients one of the most recognized benefits of treatment with cholinesterase inhibitors, including donepezil, is stabilization and even temporary improvement in attention performance [51, 52]. Since donepezil effectively and preferentially normalized EEG rhythmicity in the 2–12 Hz frequency in AD patients [53, 54], a frequency range which also covers absence seizure discharges, it might be assumed that reduction of these pathological events directly contributes to attention improvement in donepezil-treated patients. However, clinical data show inconsistency in the effectiveness of donepezil treatment since in a number of AD patients both the presence of cortical EEG oscillatory abnormalities and worsening of cognitive symptoms were described despite the prolonged use of the drug [55]. Interestingly, in these “nonresponders” to donepezil, resting (eye-closed) EEG during the awake state showed characteristic activity with intrusions of spindles, whose frequency greatly overlaps HVS discharges [56].

Under the freely behaving condition we found that donepezil treatment had the opposite effects on long-range cortical oscillatory connectivity in aged TgF344-AD and WT rats. Specifically, donepezil increased frontal-occipital phase-locking in TgF344-AD rats, and decreased it in age-matched WT rats. Previously, we showed that aged TgF344-AD rats had disrupted long-range connectivity when compared with their respective WT controls [13]. However, systemic administration of donepezil strengthens the connectivity between frontal and occipital oscillatory fields in transgenic rats, particularly for theta frequencies, which is important for facilitation of the formation of long-range functional networks and routing of information across cortical areas in a behaviorally relevant manner [57]. In contrast, donepezil attenuated long-range connectivity in frontal-occipital networks in WT controls. The latter result is in accordance with a recent report [58] where, using simultaneous EEG-fMRI recordings in healthy humans, it was shown that donepezil induces reduction in functional connectivity when compared with placebo.

## Conclusions

Taken together, these results imply that pharmacologically enhancing cholinergic tone with donepezil could partially reverse oscillatory abnormalities in TgF344-AD rats, which is in line with its clinical effectiveness in AD patients. A systematic review of clinical data [59, 60] showed that donepezil has significant, though practically modest, effects in improving cognition and maintaining patient function over the period of 6 months to 1 year in mild to moderate AD. Our findings further demonstrate the utility of neurophysiological signals as potential translatable biomarkers for testing therapeutic strategies in AD, given the high level of analogy of disease-associated EEG abnormalities between transgenic AD models and patients. In particular, we propose EEG measures of elicited hippocampal theta oscillations and paroxysmal HVS epileptiform discharges in TgF344-AD rats as promising markers for assessing effectiveness of new drugs targeting cognitive symptoms in AD. In summary, our study suggests good translational opportunities for neurophysiological signals recorded in TgF344-AD rats, and their application could be considered in drug discovery efforts for developing therapies with disease-modifying potential.
